# Chronic intermittent hypoxia induces oxidative stress and inflammation in brain regions associated with early‐stage neurodegeneration

**DOI:** 10.14814/phy2.13258

**Published:** 2017-05-04

**Authors:** Brina Snyder, Brent Shell, J. Thomas Cunningham, Rebecca L. Cunningham

**Affiliations:** ^1^ Institute for Health AgingUniversity of North Texas Health Science CenterFort WorthTexas; ^2^ Institute for Cardiovascular and Metabolic DiseaseUniversity of North Texas Health Science CenterFort WorthTexas

**Keywords:** Entorhinal cortex, hippocampus, RVLM, solitary tract nucleus, substantia nigra

## Abstract

Sleep apnea is a common comorbidity of neurodegenerative diseases, such as Alzheimer's disease (AD) and Parkinson's disease (PD). Previous studies have shown an association between elevated oxidative stress and inflammation with severe sleep apnea. Elevated oxidative stress and inflammation are also hallmarks of neurodegenerative diseases. We show increased oxidative stress and inflammation in a manner consistent with early stages of neurodegenerative disease in an animal model of mild sleep apnea. Male rats were exposed to 7 days chronic intermittent hypoxia (CIH) for 8 h/day during the light period. Following CIH, plasma was collected and tested for circulating oxidative stress and inflammatory markers associated with proinflammatory M1 or anti‐inflammatory M2 profiles. Tissue punches from brain regions associated with different stages of neurodegenerative diseases (early stage: substantia nigra and entorhinal cortex; intermediate: hippocampus; late stage: rostral ventrolateral medulla and solitary tract nucleus) were also assayed for inflammatory markers. A subset of the samples was examined for 8‐hydroxydeoxyguanosine (8‐OHdG) expression, a marker of oxidative stress‐induced DNA damage. Our results showed increased circulating oxidative stress and inflammation. Furthermore, brain regions associated with early‐stage (but not late‐stage) AD and PD expressed oxidative stress and inflammatory profiles consistent with reported observations in preclinical neurodegenerative disease populations. These results suggest mild CIH induces key features that are characteristic of early‐stage neurodegenerative diseases and may be an effective model to investigate mechanisms contributing to oxidative stress and inflammation in those brain regions.

## Introduction

Sleep apnea is a comorbidity of several neurodegenerative diseases, such as Alzheimer's disease (AD) (Beebe and Gozal 2002; Gagnon et al. 2014; Troussiere et al. 2014) and Parkinson's disease (PD) (Arnulf et al. 2002). Sleep apnea occurs 2–3 times more frequently in men than women, and the prevalence of sleep apnea increases with age (Young et al. 1993; Duran et al. 2001). Interestingly, in patients with PD who suffer from sleep disordered breathing, the severity of sleep apnea corresponds with the severity of PD (Diederich et al. 2005). This suggests sleep apnea may contribute to the risk of neurodegeneration.

Sleep apnea is a common disorder resulting in interruptions in breathing that cause alternating periods of reduced oxygen inspiration (apnea or hypopnea) followed by rapid reoxygenation while a patient sleeps (Dempsey et al. 2010). The reduction in oxygen inspiration leads to hypoxia, hypercapnia, and decreased intrathoracic pressure (Dick et al. 2007). The periodic rapid reoxygenation events may contribute to the pathogenesis of sleep apnea (Kaczmarek et al. 2013; Boroujerdi and Milner 2015). The severity of sleep apnea is often determined by the apnea/hypopnea index (AHI), which measures the number of times per hour a hypoxic event occurs during sleep (Ruehland et al. 2009). The AHI classification of mild, moderate, or severe sleep apnea is 5, 15, and 30 events per hour, respectively.

Recent studies have shown hypoxic events can be either neuroprotective or neurotoxic depending on the severity, frequency, and duration of the hypoxia (Peers et al. 2009; Jackman et al. 2014; Kim et al. 2014; Rosenzweig et al. 2014; Boroujerdi and Milner 2015). Acute sustained hypoxic events, defined by occurrences lasting less than 4 h, appear to precondition cells to subsequent insults by upregulating protective pathways within neurons (Stowe et al. 2012; Boroujerdi and Milner 2015; Parmar and Jones 2015). In contrast, chronic intermittent hypoxia (CIH), which occurs repeatedly during sleep every night, has been associated with increased neurodegeneration in a variety of brain nuclei (Gozal et al. 2001, 2003; Xu et al. 2004; Peng et al. 2014), which can impact different functions, such as cognition, motor ability, and homeostatic functions (Xu et al. 2004; Cunningham et al. 2012; Smith et al. 2013; Peng et al. 2014). Increased oxidative stress and inflammation, key characteristics of neurodegenerative diseases (Jenner 2003; Barnham et al. 2004; Reale et al. 2008, 2009), are observed in sleep apnea (Lavie 2014; May and Mehra 2014).

Age‐associated oxidative stress may be a major contributor to many age‐related diseases. Elevated oxidative stress is implicated in the progression of neurodegeneration by leading to protein dysfunction and a resultant inflammatory environment within cells (Barnham et al. 2004; Dai et al. 2014; Mittal et al. 2014). Previous studies in our laboratory have demonstrated oxidative stress can induce neuroinflammation which, in turn, can elevate oxidative stress above a threshold which is neurotoxic to cells and leads to neuronal apoptosis (Holmes et al. 2016) and is disrupted by inhibiting inflammatory pathway components.

One major component of the inflammatory system is macrophages. Within the central nervous system (CNS), macrophages and microglia are activated by cytokines to differentiate into either a proinflammatory (M1) or an anti‐inflammatory (M2) profile (Du et al. 2016; Komohara et al. 2016). Cytokines such as IL‐6, IL‐5, TNF‐*α*, and IFN‐*γ* are classically responsible for activation of microglia into the proinflammatory M1 profile to remove pathogens and damaged tissue, as well as activate apoptotic pathways. Alternatively, IL‐4, IL‐10, and IL‐13 cytokines allow for microglial differentiation into anti‐inflammatory M2 profiles to promote cell survival and inhibit inflammatory pathways (Table 1). Activated microglia then release cytokines consistent with their profile to further induce additional immune responses. Under homeostatic conditions, these activated profiles are balanced to maintain cellular health (Du et al. 2016). However, dysregulation of these complementary profiles can caused by chronic activation of the inflammatory system and is associated with detrimental effects (Hirata et al. 2011; Zhang et al. 2012; Perry and Holmes 2014; He et al. 2015; Ramani et al. 2015; Komohara et al. 2016) in many disease states. An increase in proinflammatory M1 cytokines has been observed in neurodegeneration (Du et al. 2016). These same M1 cytokines are also elevated in patients with sleep apnea (Gozal and Serpero 2008; Smith et al. 2013; May and Mehra 2014) and in animal models (Sapin et al. 2015; Smith et al. 2013). Chronic activation of proinflammatory M1 markers associated with sleep apnea may be a contributing risk factor to neurodegenerative diseases (Lucas et al. 2006; Chao et al. 2014; Monson et al. 2014).

**Table 1:** Cytokines of interest for this study and known role in macrophage recruitment

Cytokines of interest			
Microglia profile	Cytokine	Physiological effects	References
M1	IL‐6	Proinflammatory	Bamberger and Landreth (2002)
	TNF‐*α*	Pathogen removal	Perry and Holmes (2014)
	IFN‐*γ*	Apoptotic events	Sierra et al. (2007)
M2	IL‐10	Anti‐inflammatory	Varnum and Ikezu (2012)
	IL‐4	Prosurvival signals	Du et al. (2016)
	IL‐13	Wound healing	

IL, interleukin; TNF, tumor necrosis factor; IFN, interferon.

The rodent model of CIH allows for the study of hypoxia‐reoxygenation mechanisms, similar to those experienced by patients with sleep apnea (Fletcher et al. 1992). Models of severe CIH have been documented to mimic the hypoxia experienced by patients with severe sleep apnea (AHI>30), as well as elevated inflammation associated with neurodegenerative diseases (Gozal et al. 2001; Smith et al. 2013; Kim et al. 2014). However, few studies have examined the impact of mild hypoxia (AHI < 30) on inflammation or oxidative stress within specific regions of the brain. We hypothesize mild CIH increases oxidative stress and inflammation in brain regions affected by early‐stage neurodegenerative diseases.

To examine the role of sleep apnea on oxidative stress and neuroinflammation in brain areas at risk for neurodegeneration, we exposed male rats to CIH (AHI = 10), a model of mild sleep apnea. Five brain nuclei that have been shown to be affected by neurodegenerative diseases at different stages of progression were selected and assessed for changes in oxidative stress and M1/M2 activating cytokine expression. Brain nuclei known to be affected during early‐stage neurodegeneration are the substantia nigra (SN) in PD (Crocker 1997; Braak et al. 2003) and the entorhinal cortex (ETC) in AD (Braak et al. 2006; Mayeux and Stern 2012). Neurodegeneration is observed in the hippocampus during intermediate stages and is believed to be a leading contributor to the dementia experienced by patients with AD (Reitz and Mayeux 2014). The rostral ventrolateral medulla (RVLM) and the solitary tract nucleus (NTS) are affected during advanced late‐stage neurodegenerative diseases, and play a major role in maintaining homeostatic functions (Allen et al. 2006; Cunningham et al. 2012; Wu et al. 2012; Menani et al. 2014). Our results show significant elevations in oxidative stress and proinflammatory M1 cytokines in the SN and ETC, regions associated with early‐stage neurodegeneration. This is the first study we are aware of to characterize the inflammatory profile of the SN and brainstem regions in response to mild CIH.

## Methods

### Animals

All experiments were conducted according to the National Institutes of Health guidelines on laboratory animals and approved by the Institutional Care and Use Committee at UNT Health Science Center. Thirty‐seven adult male Sprague Dawley rats (250–300 g body weight, Charles River) were individually housed in a temperature‐controlled environment with the lights on a 12:12‐h cycle. Food and water were provided ad libitum. Male rats were used because sleep apnea is more prevalent in men than women (Lee and Gilbert 2016).

### Chronic intermittent hypoxia

One week after arrival, rats were separated into either normoxia (*n* = 13) or CIH (*n* = 18) treatment groups. Rats were placed into custom‐built Plexi glass chambers to acclimate to the CIH apparatus for 1 week at normoxic conditions (21% oxygen) as described previously (Cunningham et al. 2012). Acclimation to the chambers was followed by CIH exposure for 7 days from 8 am to 4 pm during the light (sleep) cycle. Oxygen concentrations were controlled by timers, which alternate the flow of room air and nitrogen into the chambers. Our CIH protocol involves 6 min cycles of low oxygen (10%) followed by reoxygenation (21%) for 8 h during the light phase to model an AHI of 10 as described previously (Knight et al. 2011). This protocol is used to model the frequency of hypoxic episode experienced by patients with mild sleep apnea (Epstein et al. 2009). Specifically, nitrogen is injected into the chamber for 90 sec and is titrated so that 10% oxygen occurs within each chamber a few seconds after nitrogen injection halts. Over a period of 90 sec, no gas is injected and the oxygen concentration begins to slowly rise. Room air is then infused into the chamber for a total of 90 sec, which returns the oxygen levels to 21% within 30 sec. Normal room oxygen levels are then maintained for an additional 90 sec. This cycle repeats throughout the 8‐h treatment time. For the remaining 16 h, animals were exposed to room air. To control for sleep deprivation due to noises from the CIH apparatus, normoxic controls were housed under similar conditions but were not exposed to hypoxia.

### Sample collection

Within 16 h of the final CIH exposure, during the first 2 h of the light phase, animals were deeply anesthetized (inactin, 100 mg/kg i.p.) and sacrificed as described previously (Knight et al. 2011). Blood was collected in 7 mL EDTA tubes. The samples were then centrifuged at 2240*g* for 10 min at 4°C. Plasma was removed and stored in microcentrifuge tubes at −80°C until assayed.

Brains were immediately removed upon euthanasia, flash frozen in PBS on dry ice, and then sliced into 1‐mm coronal sections using a commercially available matrix (ASI Instruments, Warren MI). Brain nuclei containing the rostral ventrolateral medulla (RVLM) (−11.80 mm from Bregma), solitary tract nucleus (NTS) (−13.68 mm from Bregma), substantia nigra (SN) (−5.30 mm from Bregma) or entorhinal cortex (ETC) (−5.30 mm from Bregma), and dorsal hippocampus (−4.52 mm from Bregma) were isolated according to Paxinos and Watson's The Rat Brain Stereotaxic Coordinates (Paxinos 1998) using blunt 20‐gauge needles attached to 1 mL syringes, immediately frozen on dry ice in 1.5 mL microcentrifuge tubes, then stored at −80°C until homogenized for assays.

### Tissue homogenization

Each sample was incubated in 50 *μ*L RIPA lysis buffer (Amresco) with 3 *μ*mol/L phosphatase inhibitor (Sigma‐Aldrich) and 1 *μ*mol/L dithiothreitol (Sigma‐Aldrich), then incubated on ice prior to sonication three times. Samples were then centrifuged 20 min at 12,000*g* at 4°C. The supernatant was extracted and transferred to a clean 1.7 mL microcentrifuge tube. Protein quantification was assessed using the modified Lowry protein assay kit (Thermo Scientific), and homogenate was stored at −80°C until used in multiplexing protocols.

### Advanced oxidative protein products assay

Circulating oxidative stress was assayed using Cell Biolabs, Inc., OxiSelect AOPP assay kit, according to our previously published protocol (Cunningham et al. 2011). This kit measures the amount (*μ*mol/L) of all oxidized proteins in the sample relative to a known standard. Chloramine in the kit reacts with oxidized proteins to produce a color change which can be read at 340 nm.

### Inflammatory panel

IL‐6, TNF‐*α*, IFN‐*γ*, and IL‐5 protein levels were used as indicators of proinflammatory M1 macrophage recruitment, while IL‐10, IL‐13, and IL‐4 protein levels were used as indicators of anti‐inflammatory M2 macrophage activation. Cytokine levels in brain tissue homogenate samples were quantified using Proinflammatory Panel 1 (rat) V‐PLEX kit from Meso Scale Diagnostics (Tables 2, 3, 4). This immunoassay allows for quantification (pg/mL) of multiple analytes from a single sample using a sandwich ELISA protocol with each analyte of interest located in a specific region of each well. Using the V‐PLEX assay, 120 *μ*g of each tissue sample was quantified. Plasma cytokine levels were measured using Biorad's Bioplex Rat Th1/Th2 12‐Plex kit and fluorescence measured on a Luminex platform (MAGPIX, Luminex Corporation, Austin, TX). Plasma samples (diluted 1:4) were loaded into each well of a 96‐well pate and capture antibodies attached to magnetic beads reacted with each analyte of interest within each sample on a different region of the bead. This immunoassay quantifies the amount (pg/mL) of each cytokine relative to a known standard using Bio‐Plex Manager Software 6.1. Quantities of each cytokine were normalized to normoxic controls. To analyze the effects of CIH on proinflammatory M1 (IL‐5, IL‐6, TNF‐*α*, & IFN‐*γ*) and anti‐inflammatory M2 (IL‐4, IL‐10, & IL‐13) cytokines as a group, individual cytokine values were summed together to create a composite value.

**Table 2:** Cytokines per region implicated in early‐stage neurodegeneration

Cytokine	Substantia nigra		Entorhinal cortex	
	CIH (*n* = 8)	*P*	CIH (*n* = 16)	*P*
IL‐10	110.63 ± 15.17	0.592	81.75 ± 10.99	0.240
IL‐13	102.52 ± 16.22	0.910	102.27 ± 7.11	0.878
IL‐4	142.27 ± 55.80	0.525	93.42 ± 13.45	0.797
IL‐6	154.78 ± 14.99	0.023^a^	116.01 ± 8.49	0.217
TNF‐*α*	148.73 ± 18.44	0.052^a^	93.66 ± 10.20	0.697
IFN‐*γ*	105.61 ± 7.11	0.569	103.70 ± 5.53	0.691
IL‐5	92.80 ± 13.73	0.635	114.81 ± 14.29	0.472

Values are expressed as percent of control (pg/mL) ± SEM. IL, interleukin; TNF, tumor necrosis factor; IFN, interferon.

a *P* < 0.05.

**Table 3:** Cytokines per region implicated in intermediate‐stage neurodegeneration

Cytokine	Hippocampus	
	CIH (*n* = 16)	*P*
IL‐10	102.88 ± 18.81	0.919
IL‐13	99.00 ± 19.90	0.784
IL‐4	100.13 ± 23.10	0.621
IL‐6	85.08 ± 11.47	0.228
TNF‐*α*	87.62 ± 16.46	0.997
IFN‐*γ*	89.16 ± 11.44	0.316
IL‐5	103.43 ± 7.19	0.814

Values are expressed as percent of control (pg/mL) ± SEM. IL, interleukin; TNF, tumor necrosis factor; IFN, interferon.

**Table 4:** Cytokines per region implicated in late‐stage neurodegeneration

Cytokine	Solitary tract nucleus		Rostroventrolateral medulla	
	CIH (*n*=8)	*P*	CIH (*n*=16)	*P*
IL‐10	58.60 ± 14.97	0.171	70.96 ± 9.77	0.051^a^
IL‐13	51.63 ± 13.96	0.404	75.45 ± 12.97	0.163
IL‐4	63.13 ± 15.20	0.292	54.96 ± 10.93	0.005^a^
IL‐6	70.29 ± 16.54	0.411	75.62 ± 9.05	0.037^a^
TNF‐*α*	65.09 ± 17.17	0.226	69.93 ± 9.53	0.043^a^
IFN‐*γ*	74.67 ± 9.72	0.106	73.22 ± 7.01	0.019^a^
IL‐5	71.80 ± 13.69	0.344	77.04 ± 16.47	0.464

Values are expressed as percent of control (pg/mL) ± SEM. IL, interleukin; TNF, tumor necrosis factor; IFN, interferon.

a *P*<0.05.

### Immunohistochemistry

A subset of rats exposed to either CIH (*n* = 3) or normoxic (*n* = 3) conditions were transcardially flushed with 0.1 mol/L PBS, and then perfused with 4% paraformaldehyde in 0.1 mol/L PBS following 7 days CIH exposure. Brains were removed from the skull and stored overnight in 4% paraformaldehyde at 4°C. Twenty‐four hours later, brains were transferred to 30% sucrose in PBS at 4°C for 3 days, then sliced on a cryostat (Thermo Scientific, CryoStar NX70) into 40‐*μ*m coronal sections containing the regions of interest as described previously (Cunningham et al. 2012). Three separate sets of coronal sections containing the SN, ETC, and dorsal hippocampus (−4.80 to −6.04 mm from Bregma), RVLM (−11.30 to −12.72 mm from Bregma), or NTS (−13.68 to −14.08 mm from Bregma) were collected from each brain and stored in cryoprotectant at −20°C until they were processed for immunofluorescence (Watson et al. 1986).

One set of sections from each animal was rinsed in PBS and incubated in PBS in 3% horse serum for 4 h. The sections were rinsed and incubated for 72 h at 4°C in primary goat 8‐hydroxydeoxyguanosine (8‐OHdG) antibody (Abcam ab10802, 1:10,000). Afterward, the sections were rinsed in PBS and incubated in secondary antibody (Alexa Fluor 488 goat anti‐rabbit 1:2000; Abcam ab150105) overnight at 4°C. Sections were then rinsed in PBS, mounted on slides, and sealed using Vectashield mounting medium containing DAPI. Slides were stored at 4°C until imaged.

Images of DAPI and 8‐OHdG immunofluorescence were captured from each section using an inverted fluorescence microscope (VWR International) equipped with a digital camera (Coolsnap MYO, Photometrics, Tucson, AZ). The numbers of 8‐OHdG/DAPI+ cells per image were quantified using NIS Elements Imaging Software 4.50 (Nikon Instruments, Inc., Melville, NY).

### Statistical analysis

Assay results are reported as percent of control (individual value/[average of control values] × 100). IBM SPSS (SPSS v. 23, IBM, 2015) was used for statistical analysis. Independent *t*‐tests assessed the difference between the means of advanced oxidative protein products (AOPP), cytokines, and 8‐OHdG expression by treatment. Pairwise *t*‐tests were used to investigate the difference between M1 and M2 cytokine expression within animals exposed to CIH. Results are shown as mean ± SEM. Statistical significance was at *P* < 0.05.

## Results

### Exposure to CIH increases circulating oxidative stress

**Figure 1:**
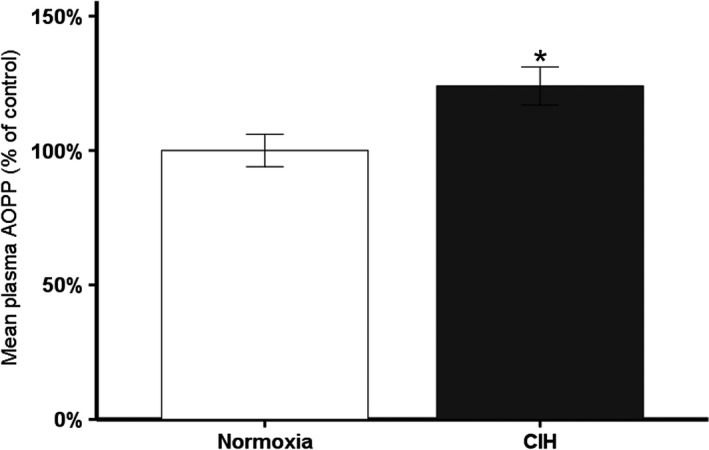
Plasma AOPP levels of animals exposed to CIH: 7 days exposure to CIH (*n* = 18) significantly increases circulating oxidative stress in male rats. *P ≤ 0.05.

One hallmark of neurodegenerative diseases is elevated oxidative stress (Barnham et al. 2004). Circulating AOPP levels were measured as a marker of oxidative stress. Plasma from animals exposed to CIH exhibited an average of 24% more oxidative stress than control animals, which was statically significant (Fig. 1; *t*(28) = −2.481; *P* < 0.05). These results show that mild CIH increases systemic oxidative stress, consistent with the elevation in oxidative stress observed in patients with neurodegenerative diseases (Dai et al. 2014).

### Exposure to CIH increases oxidative stress in brain regions associated with neurodegenerative diseases

**Figure 2:**
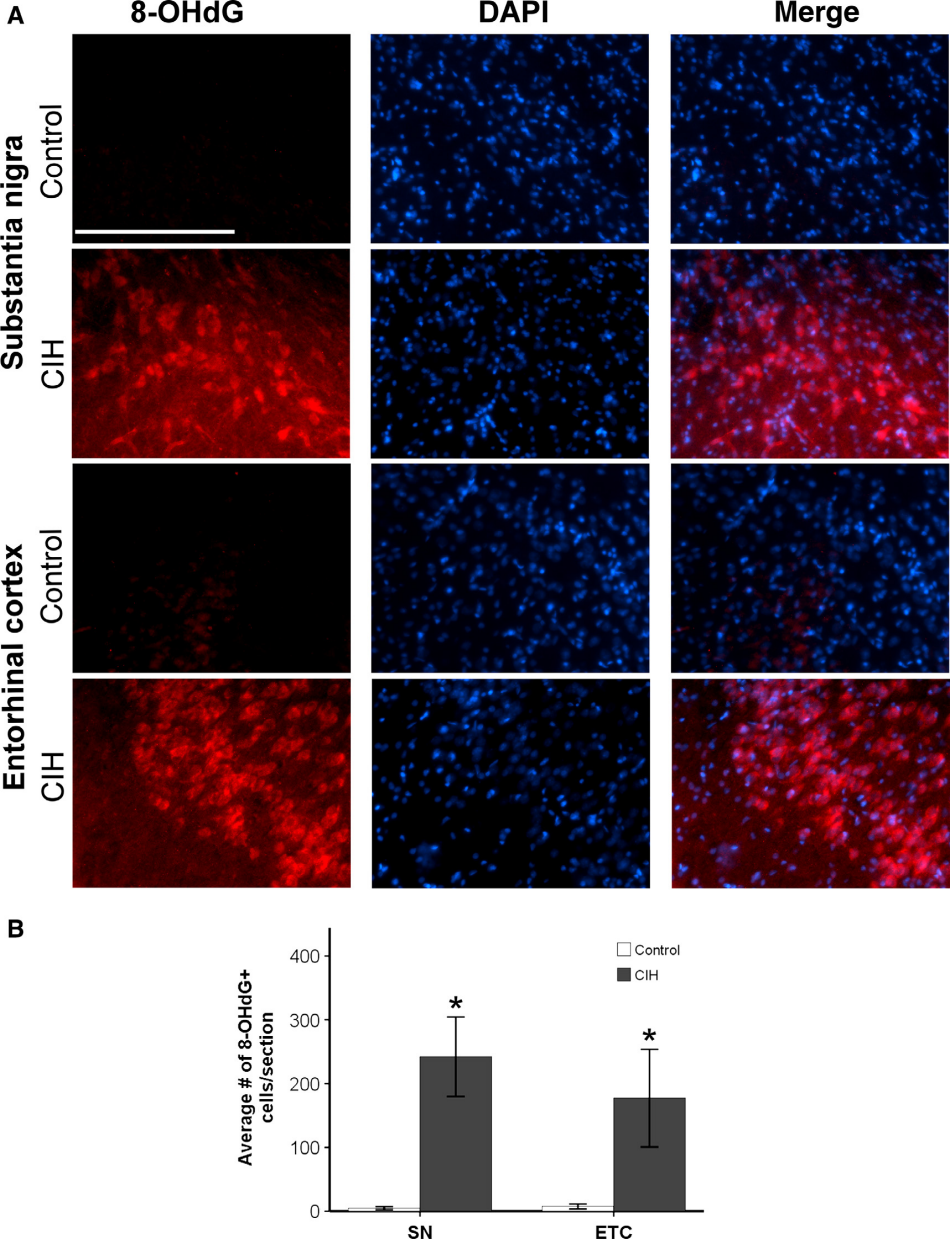
Significantly elevated expression of 8‐OHdG was observed in brain regions associated with early‐stage neurodegenerative diseases following 7 days of CIH exposure. *P ≤ 0.05. (A) An elevation of oxidative stress in animals exposed to 7 days CIH is observed in SN and layer II of the ETC as compared to controls animals. (B) CIH increased oxidative stress in the SN and ETC layer II. Data presented as mean number of cells exhibiting 8‐OHdG immunoreactivity/section ± SEM. Scale bar = 100 *μ*m.

**Figure 3:**
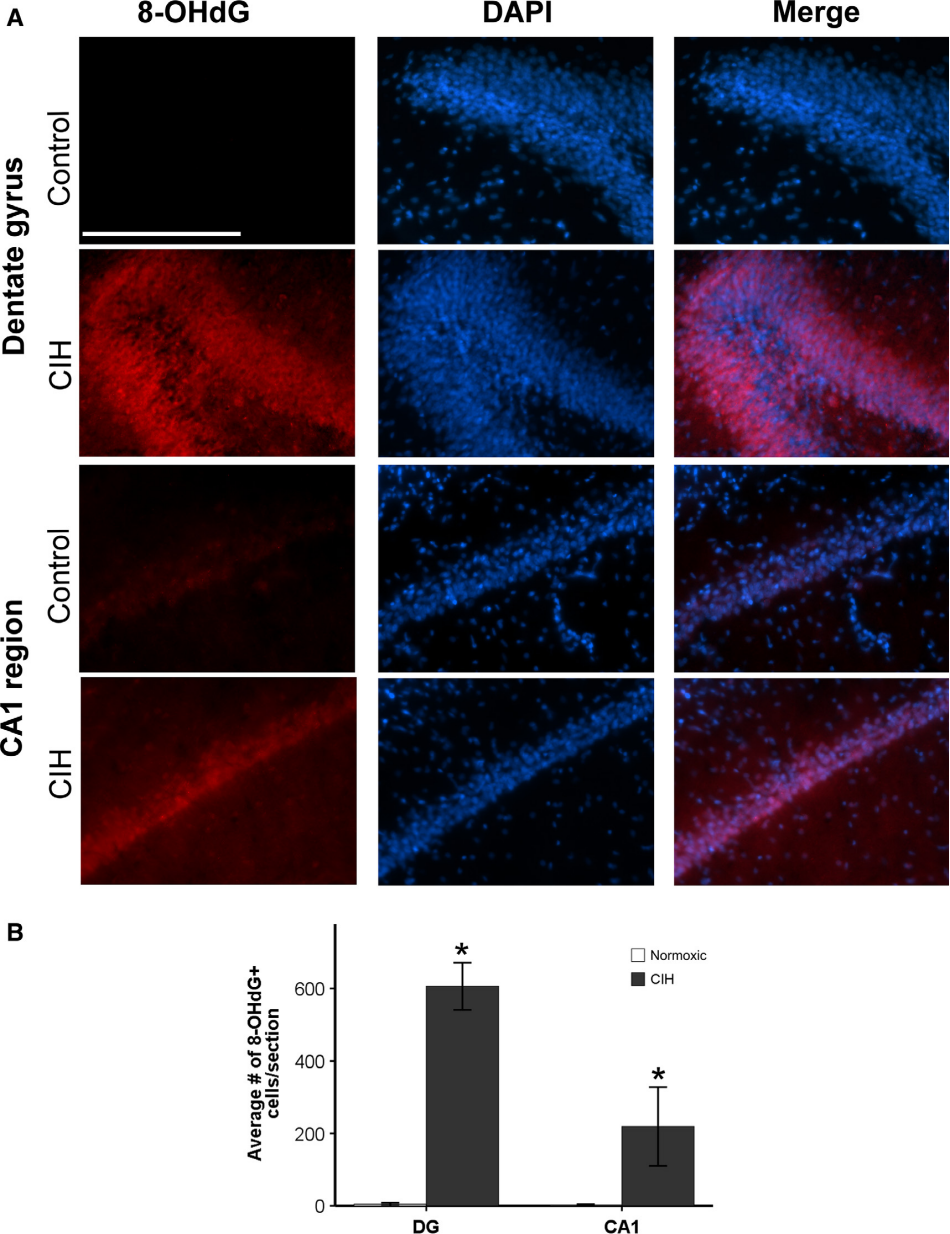
Significantly elevated expression of 8‐OHdG was observed in brain regions associated with intermediate AD following 7 days of CIH exposure. *P ≤ 0.05. (A) An elevation of oxidative stress in animals exposed to 7 days CIH is observed in both the dentate gyrus and the CA1 regions of the hippocampus. (B) CIH increased oxidative stress in the CA1 and dentate gyrus (DG). Data presented as mean number of cells exhibiting 8‐OHdG immunoreactivity/section ± SEM. Scale bar = 100 *μ*m.

To examine if CIH induces oxidative stress in the brain, we quantified 8‐OHdG protein expression. 8‐OHdG is a marker of oxidative stress‐induced DNA damage. CIH was associated with significant oxidative stress‐mediated DNA damage in the SN, layer II of the ETC, and dorsal hippocampus, brain regions linked with PD and AD, respectively (Figs. 2A and 3A). The number of cells with oxidative stress‐induced DNA damage following CIH exposure was significantly greater in the SN (*t* = −4.436; *P* < 0.05), the ETC (*t* = −2.796; *P* < 0.05), the dentate gyrus (*t* = −11.620; *P* < 0.05), and the CA1 regions of the hippocampus (*t* = −2.515; *P* < 0.05) (Figs. 2B and 3B). No 8‐OHdG immunoreactivity was observed in the RVLM or NTS (data not shown), indicating that mild CIH does not increase oxidative stress‐induced DNA damage to brain regions affected during late‐stage neurodegenerative diseases. This evidence supports our hypothesis that mild CIH elevates oxidative stress in brain regions linked with early‐ to intermediate‐stage neurodegeneration, but not late‐stage neurodegeneration.

### Alterations in the M1/M2 cytokine profiles are observed in animals exposed to CIH

**Figure 4:**
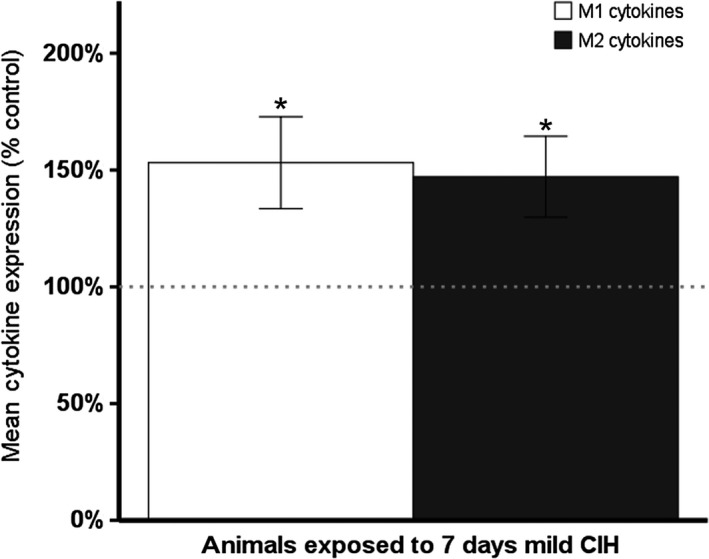
CIH induces an increase in circulating M1 (IL‐6, TNF‐*α*, IFN‐*γ*, IL‐5) and M2 (IL‐4, IL‐10, IL‐13) inflammatory markers. Dotted line indicates normalized controls (normoxia = 13, CIH = 18). *P ≤ 0.05.

CIH induced a mild peripheral inflammatory response (Fig. 4) similar to the elevated inflammation observed in patients and animal models of advanced neurodegenerative diseases (Reale et al. 2008, 2009; Zhang et al. 2013; Chao et al. 2014). Both M1 (*t*(31) = −2.431; *P* < 0.05) and M2 (*t*(31) = −2.456; *P* < 0.05) circulating cytokines were significantly elevated after CIH compared to control rats under normoxic conditions.

**Figure 5:**
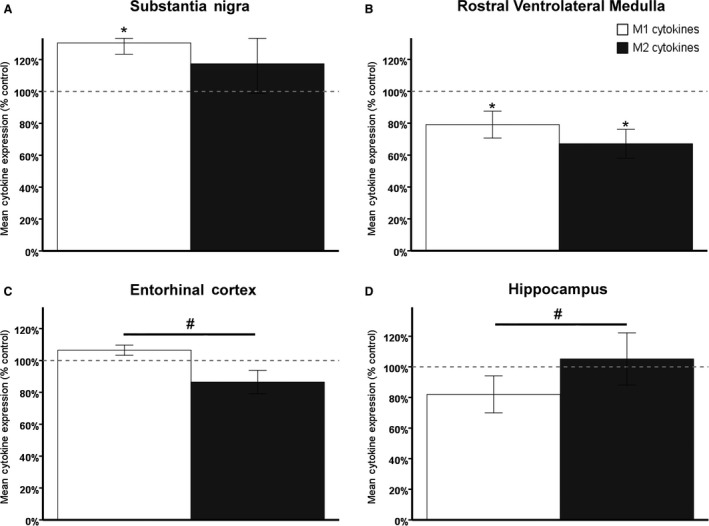
Region‐specific inflammatory responses to CIH are observed in the brain, resulting in a proinflammatory profile in the SN and ETC. An anti‐inflammatory profile is observed in the dorsal hippocampus. Dotted line indicates normalized controls. * indicates significant compared to control, # indicates significant difference within animal. (A) A significant elevation of M1 cytokines in the SN of animals exposed to CIH leads to a higher M1 cytokine profile than M2 profile in that brain region (normoxia = 12, CIH = 15). (B) An overall reduction in M1 and M2 cytokines is observed in the RVLM of animals exposed to CIH. No significant difference in the M1/M2 cytokine profile was observed in this region associated with late‐stage neurodegenerative diseases (normoxia = 13, CIH = 15). (C) M2 cytokines are significantly lower than M1 cytokines in the ETC of animals exposed to CIH, which elevates the M1 profile over the M2 profile (normoxia = 11, CIH = 9). (D) M1 cytokines are significantly lower than M2 cytokines in the HIPP following CIH (normoxia = 13, CIH = 15).

Interestingly, inflammatory profiles in the CNS were altered in a brain region specific manner. Differences in cytokine expression due to CIH were observed in several regions (Fig. 5, Tables 2, 3, 4). In the SN, an area associated with early‐stage PD, proinflammatory M1 cytokines, but not anti‐inflammatory M2 cytokines, were significantly elevated in animals exposed to CIH (Fig. 5A; *t*(25) = −3.076; *P* < 0.05, Table 2). In the RVLM, which is implicated in later stages of neurodegenerative diseases, CIH was associated with significant decreases in both M1 and M2 cytokines compared to normoxic controls (Fig. 5B; M1: *t*(26) = 2.055; M2: *t*(26) = 2.600; both *P* < 0.05, Table 4).

A different pattern was observed in regions affected by AD. In the ETC, a region associated with early‐stage AD, proinflammatory M1 cytokines were significantly elevated over anti‐inflammatory M2 cytokines within each animal exposed to CIH (Fig. 5C; *t*(8) = 2.379, *P* < 0.05, Table 2). Within the dorsal hippocampus, M2 cytokines were significantly elevated compared to M1 cytokines within animals exposed to CIH (Fig. 5D; *t*(14) = −2.337; *P* < 0.05, Table 3). No differences were observed in the NTS (data not shown).

## Discussion

Mild CIH induces effects in brain regions consistent with early‐ to intermediate‐stage neurodegenerative disease. Patients with preclinical AD exhibit elevated systemic oxidative stress and inflammation (Migliore et al. 2005; Reale et al. 2008). Pathological protein aggregates associated with AD appear in the second layer of the ETC during early stages (Khan et al. 2014; Braak and Del Tredici 2015), and then progress to the hippocampus during intermediate stages when AD symptoms begin to manifest (Braak and Braak 1994; Braak et al. 2006; Mayeux and Stern 2012). During later stages, those effects spread to cortical and brainstem areas, and are associated with a higher severity of symptoms as well as a loss of autonomic functions (Braak and Braak 1994; Braak et al. 2006). Similarly, patients with early‐stage PD exhibit elevated systemic oxidative stress, inflammation, and protein aggregates in the SN (Braak et al. 2003; Migliore et al. 2005; Reale et al. 2009). These systemic effects have been observed in rodent models of severe CIH (Gozal and Serpero 2008; Lavie 2014; May and Mehra 2014). Severe sleep apnea has been established as a comorbidity of several neurodegenerative diseases (Arnulf et al. 2002; Ancoli‐Israel et al. 2008) and is associated with elevated oxidative stress and inflammation. Recent studies (Sheu et al. 2015; Yeh et al. 2016) indicate men with sleep apnea are at higher risk to develop PD and the severity of sleep apnea increases with the severity of PD (Diederich et al. 2005). This suggests sleep apnea may be one contributor to the risk of developing a neurodegenerative disease. While clinical treatment of patients with moderate/severe sleep apnea is considered standard, treatment of mild sleep apnea is optional (Kushida et al. 2006; Epstein et al. 2009). Our results with mild CIH suggest that mild sleep apnea may be a risk factor for neurodegeneration.

Our results show that mild CIH elevates oxidative stress both peripherally and centrally in a pattern consistent with early‐stage neurodegenerative diseases (Migliore et al. 2005). Furthermore, this effect is not contributable to sleep deprivation as it is not observed in the normoxic controls. Oxidative stress has been implicated in AD and PD progression, with detrimental effects observed in the SN, ETC, and hippocampus during the early and intermediate stages of these diseases (Barnham et al. 2004; Migliore et al. 2005). The number of cells in these regions exhibiting DNA damage due to oxidative stress was elevated in our model of mild CIH (Figs. 2 and 3). This may be a precursor to the hippocampal damage observed in longer and more severe models of CIH (Sapin et al. 2015; Gozal et al. 2001, 2003; Xu et al. 2004). No oxidative stress‐induced DNA damage was observed in areas associated with late‐stage neurodegenerative disease symptoms. It is conceivable that longer exposure to mild CIH may contribute to an environment in which damage to later stage regions occurs.

Our results also demonstrate inflammation consistent with early‐stage neurodegenerative diseases. An overall increase in circulating inflammatory markers is observed in animals exposed to CIH (Fig. 4) similar to the rise in inflammatory markers observed in patients with neurodegenerative diseases (Bamberger and Landreth 2002; Perry and Holmes 2014; Du et al. 2016). Prior studies have shown specific brain regions exhibit different inflammatory responses to models of intermittent hypoxia. These responses are dependent on the severity of the model and whether the exposure to intermittent hypoxia is chronic or acute (Gozal et al. 2003; Row et al. 2007; Smith et al. 2013).

In our model of CIH, we also observed regionally different responses in inflammatory markers (Fig. 5) consistent with previously published observations (Sapin et al. 2015; Smith et al. 2013). Microglial differentiation is complex and guided by a variety of signals dependent on the immediate needs of the specific region. Expression of either M1 proinflammatory markers or M2 anti‐inflammatory markers is in response to the cytokines present within local environmental milieu (Table 1). Cytokines may be released by astrocytes or neurons in addition to other activated microglia. These cytokine signals further activate naïve microglia to respond to the needs of the cell and differentiate into either proinflammatory M1 or anti‐inflammatory M2 microglia primed to either remove damage or promote neuronal survival (Varnum and Ikezu 2012). The M1/M2 cytokine profiles observed in this study are consistent with previously published data observed in cases of early‐stage neurodegenerative disease (Du et al. 2016; Lopez Gonzalez et al. 2016).

We observed a proinflammatory profile in the SN and ETC. In the SN and the ETC, an elevation of M1 proinflammatory cytokines was observed in animals exposed to CIH. This suggests the local environment in each of these early‐stage regions is favorable to elevated M1 microglial activation and mimics the microglial response in those regions in patients with either PD (Gerhard et al. 2006) or early‐stage AD (Khan et al. 2014; Lopez Gonzalez et al. 2016). While these initial changes are mildly elevated, an increase in inflammation can further increase oxidative stress. This may be one mechanism whereby CIH switches from a neuroprotective environment to a neurotoxic one.

Interestingly, we observed an increase in the anti‐inflammatory cytokine profile in the hippocampus, which is consistent with results seen in patients during the early to intermediate stages of AD (Varnum and Ikezu 2012). This may reflect early compensatory mechanisms by microglia to attempt to protect against further damage. The overall decrease in both M1 and M2 profiles in the RVLM may also be compensatory to suppress early damage or it could be indicating that there is no inflammatory response, as no oxidative stress damage was observed in this region. No significant effects in the M1/M2 profiles were observed in the NTS. The results observed in these two regions may be due to processes which maintain homeostatic function. These results are consistent with the literature implicating these areas in late‐stage AD (Braak et al. 2006).

Oxidative stress damage in layer II of the ETC is one of the earliest observable hallmarks in AD pathology, and it occurs during stages I/II prior to clinical symptom manifestation (Terni et al. 2010; Braak and Del Tredici 2015). In PD, oxidative stress levels are elevated systemically and in the CNS during preclinical stages (Migliore et al. 2005; Nakabeppu et al. 2007; Reale et al. 2009). Mild CIH elevates oxidative stress both in the periphery and in brain regions associated with each of these diseases in a manner similar to early‐stage observations. Current efforts toward developing a biomarker panel to identify individuals during early stages of neurodegenerative disease are ongoing (Lista et al. 2015; Dubois et al. 2016). Our data along with other publications indicate that oxidative stress should be considered for inclusion in these biomarker assays (Holmes et al. 2013; Bolner et al. 2016; Wang et al. 2016).

Currently, AD and PD are not able to be diagnosed during early stages of neurodegeneration. Treatments are typically initiated once a significant amount of neurodegeneration has occurred and do not prevent progression of these types of diseases. For example, clinical AD symptoms do not appear until Braak stage V when the pathology has progressed into the hippocampus and a significant amount of neuronal loss has occurred (Braak and Braak 1994). PD is not diagnosed until there has been a loss of 80% of the dopaminergic cells within the SN (Braak et al. 2003; Lee and Gilbert 2016). Therefore, efforts have been focused toward identifying early modifiable factors to prevent neuronal loss and improve therapeutic outcomes. Our results indicate that mild CIH may be one modifiable factor and an appropriate model to study mechanisms leading to elevated oxidative stress and inflammation in early‐stage neurodegenerative disease‐associated brain regions.

While our initial observations are promising, there are several limitations to our study, such as a lack of investigation into downstream inflammatory signaling, neurodegenerative markers, and behavioral indexes. Therefore, future studies will target motor and cognitive behaviors, downstream markers of proinflammatory activation (i.e., cyclooxygenase‐2 protein expression), and traditional biomarkers of neurodegenerative diseases, such as Lewy body or tau protein accumulation.
